# Associations between 24-hour movement behaviours and psychological wellbeing in adolescents using compositional data analysis

**DOI:** 10.1186/s44167-025-00094-8

**Published:** 2025-12-20

**Authors:** Gunchmaa Nyam, Natalie Lander, Ana Maria Contardo Ayala, Claudia Strugnell, Jo Salmon

**Affiliations:** Institute for Physical Activity and Nutrition (IPAN), School of Exercise and Nutrition Sciences, Deakin University, 221 Burwood Hwy, Melbourne, Burwood, VIC 3125 Australia

**Keywords:** Compositional data analysis, Physical activity, Sedentary, Wellbeing, Adolescent health, Accelerometer

## Abstract

**Background:**

Adolescent psychological wellbeing is a critical determinant of lifelong health. Global data suggest a concerning decline in adolescent wellbeing. While the 24-hour movement behaviours, moderate to vigorous physical activity (MVPA), light physical activity (LPA), sedentary time, and sleep, have been linked to mental health outcomes; their associations with specific domains of adolescent psychological wellbeing remain underexplored. This study used compositional data analysis (CoDA) to examine how time-use relate to domain-specific wellbeing in Australian secondary school adolescents.

**Methods:**

Data were drawn from 124 adolescents (aged 13–17 years) participating in the TransformUs Secondary effectiveness trial. Wrist worn Actigraph GT9X accelerometer captured 24-hour movement behaviour over at least three valid days (≥ 16 h/day). Wellbeing was assessed using the EPOCH Measure of Adolescent Wellbeing, which includes five domains: engagement, perseverance, optimism, connectedness, and happiness. CoDA was used to examine associations between the composition of daily movement behaviours and EPOCH domains using isometric log-ratio (ILR) transformations. A compositional time reallocation analysis (30-minutes) was also performed to explore hypothetical associations with wellbeing outcomes.

**Results:**

The average daily time-use composition was 680.9 min (47.3%) sedentary time, 473.0 min (32.8%) sleep, 250.7 min (17.4%) LPA, and 35.3 min (2.5%) MVPA. Greater time spent in LPA relative to other behaviours was significantly associated with higher happiness scores (*p* <  0.05), while greater sedentary time relative to LPA was negatively associated with happiness. Reallocating 30 min of sedentary time to LPA was associated with significant increases in happiness (β =  0.128, 95% CI: 0.034, 0.222). No significant associations were found for engagement, perseverance, or optimism.

**Conclusions:**

Adolescent daily movement behaviour composition was associated with domain-specific psychological wellbeing, particularly happiness. LPA was a potential contributor to positive psychological wellbeing. These findings suggest that even modest changes in daily routines, such as replacing sedentary time and LPA, may support adolescent flourishing. Future research should confirm these findings longitudinally and employ in intervention studies.

**Supplementary Information:**

The online version contains supplementary material available at 10.1186/s44167-025-00094-8.

## Background

 Psychological wellbeing during adolescence is a critical foundation for lifelong mental and physical health [1], and defined as emotional, social, and cognitive functioning, psychological wellbeing supports an adolescents’ ability to thrive [1]. The World Health Organization’s (WHO) Adolescent Wellbeing Framework emphasises the importance of support, confidence, and healthy relationships for cultivating wellbeing [2]. Despite this, global evidence shows concerning declines in adolescent wellbeing, particularly since the COVID-19 pandemic [3]. The WHO’s Health Behaviour in School-aged Children study has reported lower life satisfaction with increasing age, especially among girls [4]. Similarly, data from over 119,000 Australian adolescents (2017–2020) showed a significant downward trend in wellbeing, with a sharp decline in 2020, particularly among female students [5]. These declines are concerning, as positive adolescent wellbeing is associated with higher resilience [6], improved emotional regulation [7], academic success [8], and lower risk of depression in adulthood [9]. Despite its importance, psychological wellbeing in adolescents remains underexplored in comparison to mental illness, particularly in terms of its determinants.

To better understand the components of adolescent psychological wellbeing, the Engagement, Perseverance, Optimism, Connectedness, and Happiness (EPOCH) framework provides a comprehensive tool for assessing positive psychological functioning specifically in adolescents [10]. The five EPOCH domains capture both hedonic (i.e., happiness, optimism) and eudemonic (i.e., engagement, perseverance, connectedness) components of wellbeing; evaluating adolescents’ ability to manage challenges and thrive [10]. Engagement refers to full immersion in activities, associated with academic success and motivation; perseverance reflects goal setting and persistence; optimism supports emotional regulation and future oriented thinking; connectedness reflects belonging and quality relationships; and happiness reflects general life satisfaction [10]. Focusing on these domains allows a more nuanced view than mental health and helps identify which aspects of wellbeing may be most sensitive to daily experiences.

Adolescent psychological wellbeing is closely related to how time is spent across the 24-hour day, particularly through movement behaviours such as physical activity, sedentary time, and sleep [11]. According to the Australian 24-hour Movement Guidelines, adolescents aged 14–17 years should accumulate at least 60 min of moderate-to-vigorous physical activity (MVPA) daily, limit recreational screen time to no more than two hours per day, and obtain 8–10 h of sleep per night [12]. These recommendations were informed by both self-reported and device-based evidence, including compositional analyses of time-use data [13]. However, adherence remains low, with fewer than 5% of Australian adolescents meeting all three guidelines, only 16% meeting physical activity recommendations, 67% meeting sleep guidelines, and less than 10% adhering to screen-time limits [14].

These low adherence rates are concerning, as these modifiable movement behaviours have been associated with mental health outcomes [15]. Sufficient MVPA is associated with improved mood, self-esteem, and social functioning [12, 15–18], whereas excessive sedentary time, especially screen-based, is linked to increased psychological distress, lower self-worth, displace restorative behaviours, and reduce social engagement [19, 20]. Adequate sleep is also critical for emotional regulation and psychological resilience [21, 22]. Experimental studies support a causal pathway from movement behaviours to improved wellbeing, with school-based interventions demonstrating that increasing physical activity and reducing sedentary time can improve adolescents’ mental health and wellbeing [23, 24], and sleep interventions improving mood and reducing anxiety [25, 26]. At the same time, these associations are likely bidirectional, as higher wellbeing may also foster healthier movement behaviours [27, 28].

Beyond these associations, theoretical frameworks provide an understanding of how movement behaviours may influence wellbeing. Theoretical models emphasise that activity and sleep regulate circadian rhythms, neuroendocrine function, and affective states, therefore influencing emotional and cognitive outcomes [29–31]. Psychosocial perspectives highlight that these behaviours also structure adolescents’ opportunities for social interaction, identity development, and coping strategies [17, 18].

From a compositional perspective, movement behaviours are codependent as time spent in one behaviour displaces time in another. While relationships between movement behaviours and mental health outcomes such as anxiety and depression are well established [27, 32], much less is known about how they relate to positive psychological wellbeing, particularly in terms of hedonic and eudaemonic views. Previous studies have also typically examined behaviours in isolation (i.e., specific activity intensities) [20, 33, 34], overlooking their interdependence. Compositional data analysis (CoDA) accounts for the relative nature of time-use data, recognising that time spent in one behaviour is meaningful in relation to time spent in others [35]. A number of studies have applied CoDA to explore the relationships between movement behaviours and depression and anxiety in adolescents [36–38] and amongst different age groups [15, 39]. However, the application of CoDA to examine associations between movement behaviours and adolescent psychological wellbeing, especially at the level of specific domains (i.e., EPOCH), are lacking. Moreover, most existing research has mainly relied on self-reported movement behaviours [18], which are susceptible to recall errors, social desirability bias, and misinterpretation of activity intensity [40].

To address these gaps, this study applied CoDA to examine the associations between device measured 24-hour movement behaviours, including MVPA, light physical activity (LPA), sedentary time, and sleep, and domain-specific psychological wellbeing using the EPOCH framework in a sample of Australian secondary school adolescents aged 13–17 years. Our study aimed to provide an understanding of how the composition of time-use relates to different domains of adolescent wellbeing. A secondary aim was to conduct a compositional time reallocation (i.e., 30 min) analysis to estimate how hypothetical changes in time spent across movement behaviours (e.g., reallocating time from sedentary time to sleep or MVPA) may be associated with changes in specific EPOCH wellbeing domains. This analysis aimed to identify how reallocations may offer the most beneficial outcomes for adolescent wellbeing to inform future intervention planning.

## Methods

### Study design and participants

This cross-sectional study used baseline data collected from August 2023 to December 2024 as part of the TransformUs Secondary effectiveness trial, details of the trial have been published elsewhere [41]. In brief, seven secondary schools from Victoria, Australia, were recruited using a targeted convenience recruitment strategy to ensure a diverse representation of schools, including variation in school type (government, independent or Catholic), geographic location (metropolitan and regional), socioeconomic status (as indicated by Socioeconomic Indexes for Areas), and school size (> 500 students enrolled). Schools were contacted via email or telephone, and those expressing interest received a Plain Language Statement and an organisational consent form. Parents/guardians provided written consent and student assent was obtained prior to participation. Students in Years 7–10 (approximately aged 13–17) were eligible for inclusion. A total of 545 students were invited, and 193 students consented to participate (35.4% participation rate). Ethics approval was obtained from Deakin University’s Human Research Ethics Committee (Project ID: 2021−269) and the Victorian Department of Education (Project ID: 2023_004712).

### Demographic characteristics

Students completed a self-report survey that included demographic questions (e.g., date of birth, gender, year level, language spoken at home, and country of birth) using the online platform Qualtrics. Age was calculated in years by subtracting the date of birth from the date of data collection and was recorded to one decimal place for accuracy. Gender was self-reported via the baseline survey, with inclusive response options provided: female, male, prefer not to say, and other. Due to the low number of participants selecting “prefer not to say” or “other” (*n* = 6), these categories were excluded from regression analyses.

### Psychological wellbeing (EPOCH)

Psychological wellbeing was assessed using the EPOCH Measure of Adolescent Wellbeing questionnaire which has demonstrated good internal consistency (Cronbach’s α = 0.74–0.92), moderate test–retest reliability (*r* = 0.23–0.71), and construct validity [10]. Students completed the survey during class time at schools using the online platform Qualtrics on tablets. Previous research has demonstrated that the five-factor correlated model of the EPOCH measure exhibits a strong fit to the data, supporting its construct validity [42–44]. The 20-item questionnaire is a positively worded self-report measure that involves both hedonic and eudaemonic views of wellbeing by encompassing five psychological traits: engagement, perseverance, optimism, connectedness, and happiness. Students rated the 20 items on a 5-point scale, with response options including “Almost never,” “Sometimes,” “Often,” “Very often,” and “Almost always.” Scores were then averaged for each of the five domains, determining the level of positive psychological functioning and emotions (Supplementary Table 1). Higher scores indicate greater wellbeing within each domain.

### Movement behaviours

Students were asked to wear an ActiGraph GT9X accelerometer (Pensacola, FL, USA) on their non-dominant wrist for seven days, 24 h a day, to measure physical activity, sedentary time, and sleep. The accelerometers were initialised using ActiLife software (version 6.13.4) at a 30 Hz sampling frequency, and raw data were downloaded in .gt3x format.

Accelerometer data were processed using the GGIR package (version 2.6-0) in R [45]. GGIR automatically performed autocalibration, detection of non-wear time, imputation of invalid data using time-matched averages across days, and sleep detection. Calibration quality was assessed using a post-calibration error threshold of 0.02 g. Participants were excluded if they failed to provide at least 16 h/day of valid wear time on at least 3 days, or if post-calibration error exceeded 10 mg [45]. Valid days began at midnight and spanned a full 24-hour period. Non-wear time was estimated based on a criterion involving standard deviation and value range of axes for 15-min moving increments in 60-min windows [46]. Given our focus on 24-hour movement behaviours, analyses were restricted to participants with valid data spanning the full 24-hour period. The analyses were restricted to participants with ≥ 3 valid wear days with at least 16 h accelerometer wear time per day and > 0 min of sleep time. Sleep was estimated using an automated algorithm based on wrist movement variability, defining sleep as the period between sleep onset and wake time [47]. Average daily physical activity and sedentary time values were calculated using age-specific, previously validated (Euclidean Norm Minus One) thresholds for use in children and adolescents: sedentary time < 35.6; light physical activity 35.6.1-201.4; moderate physical activity (MPA) 201.4–707.0; vigorous physical activity (VPA) > 707.7 [48].

### Data analysis

Data cleaning, coding, and preparation were conducted in Stata Version 18.0 (StataCorp, TX, USA). Descriptive statistics and compositional data analyses were conducted in R (version 4.4.3) using the *compositions* and *robCompositions* packages (https://CRAN.R-project.org/package=compositions; https://CRAN.R-project.org/package=robCompositions). There were no zero values in the movement behaviour variables, thus imputation was not required. Descriptive statistics were used to summarise movement behaviours, including compositional geometric means and a variation matrix to assess the co-dependence between components. In the variation matrix, values closer to 0 indicate high co-dependence between behaviours; values closer to 1 suggest lower co-dependence [35].

Associations between movement behaviours and EPOCH wellbeing domains were examined using compositional linear regression models. Prior to regression, movement data were transformed using isometric log-ratio (ILR) transformations to account for the relative nature of time-use data, specifically with pivot coordinates [49]. Four separate models were run by rotating the movement behaviour of interest within each model to assess its association with each EPOCH domain. Model assumptions (normality and homoscedasticity of residuals, and multicollinearity among covariates) were assessed and no major violations were detected. In addition, compositional time reallocation analysis was conducted to explore the estimated differences in EPOCH wellbeing outcomes resulting from reallocating 30 min between movement behaviours while holding the total time constant. The reallocation amounts were selected based on practical relevance and alignment with previous research [50]. A 30-min change was used to reflect realistic, achievable shifts in daily activity through structured interventions [50]. All models were adjusted for age, gender, and clustering at the school level. Statistical significance was set at *p* < 0.05, and R^2^ values were used to assess model fit.

## Results

**Fig. 1:**
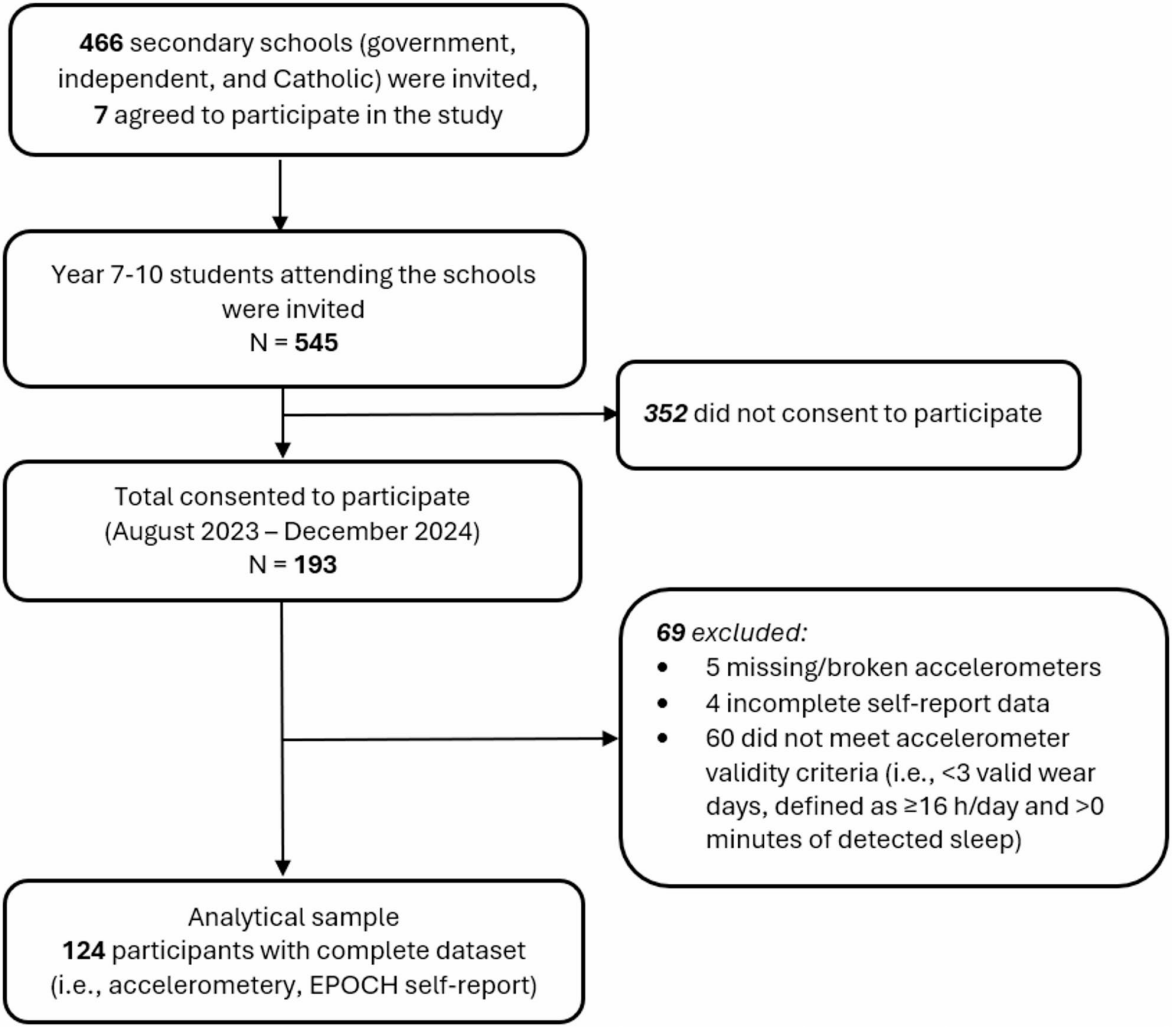
Flow chart of participants of the study

Of the 193 students who consented and were enrolled in the study, 5 (2.6%) were excluded for missing or broken accelerometers, 60 (31.1%) were excluded due to insufficient accelerometer data (fewer than three valid days [*n* = 30], less than 16 h per day valid wear [*n* = 30]), and 4 (2.1%) were excluded due to incomplete or invalid wellbeing responses (Fig. 1). Of those included in the sample, the average number of valid accelerometer days was 6.0 days (SD = 1.5), and the average daily wear time was 16.3 h (SD = 1.5), meeting inclusion thresholds for movement behaviour estimation. On average, the accelerometer data covered 68.0% of a 24-hour period.

The final analytical sample comprised of *N* = 124 participants (64.2% retention rate). Participants ranged in age from 13 to 17 years, with a mean age of 14.8 years (SD = 1.0) (Table 1). Approximately half of the sample were male (49%). Mean scores across the EPOCH domains indicated moderate to high levels of wellbeing, with the highest reported for Connectedness (4.35), followed by Happiness (3.92), Perseverance (3.62), Optimism (3.61), and Engagement (3.39).

Descriptive analysis of compositional movement behaviours indicated that almost half of the 24-h time period was spent sedentary (more than 11 h), around one-third of the time was spent in sleep (approximately 7.8 h), one-sixth of the time was spent in LPA (around 4 h), and just 2% of time was spent in MVPA (just over 30 min). Full participant characteristics and movement behaviour summaries are shown in Table 1.

**Table 1:** Descriptive characteristics of participants

Variables	Mean ± SD /*N* (%)
Age (year)	14.8 (1.0)
*Gender*	
Male	61 (49.2)
Female	57 (46.0)
Other	2 (1.6)
Prefer not to say	4 (3.2)
*Year*	
Year 7	64 (51.6)
Year 8	37 (29.8)
Year 9	22 (17.7)
Year 10	1 (0.9)
*Country of birth*	
Australia	99 (79.8)
Other	25 (20.2)
*Language spoken at home*	
English	101 (81.5)
Other	23 (18.5)
*EPOCH - Range (0–5) (mean)*	
Engagement	3.4 ± 0.9
Perseverance	3.6 ± 0.9
Optimism	3.6 ± 0.9
Connectedness	4.4 ± 0.8
Happiness	3.9 ± 0.7
Arithmetic mean (*)	
Sleep min/day	473.5 ± 67.3
Sedentary time min/day	681.3 ± 111.2
Light physical activity min/day	253.3 ± 67.2
Moderate-to-vigorous physical activity min/day	42.1 ± 21.6
Geometric mean (**)	
Sleep min (%)	473.0 (32.8)
Sedentary time min (%)	680.9 (47.3)
Light physical activity min (%)	250.7 (17.4)
Moderate-to-vigorous physical activity min (%)	35.3 (2.5)
Valid wear time (hrs/day)	21.5 (2.3)
Number of days with valid wear time	6.0 (1.5)

Supplementary Table 2 presents the variation matrix presents the pairwise log-ratio variances between the movement behaviours, which reflect the degree of compositional co-dependence. The variation matrix quantifies the degree of statistical co-dependence between behaviours, with lower values indicating that changes in one behaviour are more closely linked to changes in another [35]. The smallest variance was observed between sleep and sedentary time (0.054), followed by sleep and LPA (0.063), and sedentary time and LPA (0.088), indicating a relatively high co-dependence between these behaviours. In comparison, MVPA showed the highest log-ratio variances with all other behaviours, suggesting that MVPA is the most compositionally distinct component within the 24-h movement behaviour profile.

Table 2 presents the results of the compositional regression analysis using ILR transformed movement behaviour data and their associations with the five EPOCH wellbeing domains. Greater time spent in LPA relative to all other behaviours was significantly associated with higher happiness scores, while greater time spent in sedentary behaviour relative to the other behaviours was significantly associated with lower happiness scores. No significant associations were observed for MVPA or sleep relative to the other behaviours. Furthermore, there were no significant associations between movement behaviours and the other wellbeing domains of engagement, perseverance, optimism, or connectedness.

**Table 2:** Linear regression estimates of the association between 24-h movement behaviour composition and psychological wellbeing (*n* = 124)

Regression models (ILR)	β	SE	*p*-value	*R*^2^	Model *p*
*Engagement*				0.063	0.370
MVPA vs. (Sleep + SED + LPA)	− 0.067	0.200	0.739		
Sleep vs. (SED + LPA + MVPA)	− 0.209	0.557	0.708		
SED vs. (Sleep + LPA + MVPA)	− 0.711	0.716	0.323		
LPA vs. (Sleep + SED + MVPA)	0.485	0.585	0.409		
*Perseverance*				0.077	0.221
MVPA vs. (Sleep + SED + LPA)	− 0.028	0.196	0.887		
Sleep vs. (SED + LPA + MVPA)	− 0.498	0.545	0.363		
SED vs. (Sleep + LPA + MVPA)	− 1.070	0.701	0.128		
LPA vs. (Sleep + SED + MVPA)	0.633	0.573	0.272		
*Optimism*				0.052	0.506
MVPA vs. (Sleep + SED + LPA)	− 0.164	0.197	0.409		
Sleep vs. (SED + LPA + MVPA)	− 0.087	0.548	0.874		
SED vs. (Sleep + LPA + MVPA)	− 0.449	0.705	0.526		
LPA vs. (Sleep + SED + MVPA)	0.379	0.576	0.511		
*Connectedness*				0.108	0.638
MVPA vs. (Sleep + SED + LPA)	− 0.083	0.157	0.599		
Sleep vs. (SED + LPA + MVPA)	− 0.660	0.437	0.133		
SED vs. (Sleep + LPA + MVPA)	− 0.881	0.562	0.119		
LPA vs. (Sleep + SED + MVPA)	0.704	0.458	0.127		
*Happiness*				0.142	0.011
MVPA vs. (Sleep + SED + LPA)	− 0.030	0.173	0.863		
Sleep vs. (SED + LPA + MVPA)	− 0.476	0.485	0.328		
SED vs. (Sleep + LPA + MVPA)	− 1.320	0.622	0.036		
LPA vs. (Sleep + SED + MVPA)	1.000	0.505	0.049		

Table 3 presents the estimated differences in wellbeing scores associated with the reallocation of 30 min between pairs of movement behaviours. Reallocating 30 min from sedentary time to LPA was associated with a significant increase in happiness scores, whereas reallocating time from LPA to sedentary time was associated with a significant decrease in happiness scores. No other reallocations were significantly associated with engagement, perseverance, optimism, or connectedness.

**Table 3:** Reallocations of 30 min from one movement behaviour to another and its impact on wellbeing domain scores

Reallocation 30 min		Engagement β^a^ (95%CI)	Perseverance β (95%CI)	Optimism β (95%CI)	Connectedness β (95%CI)	Happiness β (95%CI)
From	To					
SED	Sleep	0.001 (− 0.002, 0.003)	0.000 (− 0.002, 0.003)	0.001 (− 0.002, 0.003)	− 0.000 (− 0.002, 0.002)	0.001 (− 0.001, 0.003)
SED	LPA	0.002 (− 0.002, 0.006)	0.003 (− 0.001, 0.007)	0.001 (− 0.003, 0.005)	0.003 (− 0.001, 0.006)	0.004 (0.000, 0.007)*
SED	MVPA	− 0.000 (− 0.008, 0.008)	− 0.010 (− 0.010, 0.005)	− 0.003 (− 0.010, 0.005)	− 0.000 (− 0.006, 0.006)	0.001 (− 0.006, 0.008)
LPA	Sleep	− 0.001 (− 0.006, 0.003)	− 0.003 (− 0.007, 0.002)	− 0.000 (− 0.005, 0.004)	− 0.003 (− 0.006, 0.001)	− 0.002 (− 0.006, 0.002)
LPA	SED	− 0.002 (− 0.006, 0.002)	− 0.003 (− 0.007, 0.001)	− 0.001 (− 0.005, 0.003)	− 0.003 (− 0.006, 0.001)	− 0.004 (− 0.007, − 0.001)*
LPA	MVPA	− 0.002 (− 0.012, 0.009)	− 0.006 (− 0.016, 0.004)	− 0.004 (− 0.014, 0.007)	− 0.003 (− 0.011, 0.005)	− 0.003 (− 0.012, 0.006)
Sleep	SED	− 0.001 (− 0.003, 0.002)	− 0.001 (− 0.003, 0.002)	− 0.001 (− 0.003, 0.002)	0.003 (− 0.002, 0.002)	− 0.001 (− 0.003, 0.001)
Sleep	LPA	0.001 (− 0.004, 0.006)	0.003 (− 0.002, 0.007)	0.000 (− 0.004, 0.005)	0.003 (− 0.001, 0.006)	0.002 (− 0.002, 0.006)
Sleep	MVPA	− 0.001 (− 0.009, 0.008)	− 0.003 (− 0.011, 0.005)	− 0.003 (− 0.011, 0.005)	− 0.000 (− 0.007, 0.006)	− 0.001 (− 0.008, 0.006)
MVPA	SED	− 0.000 (− 0.008, 0.008)	0.003 (− 0.005, 0.010)	0.003 (− 0.005, 0.010)	0.000 (− 0.006, 0.006)	− 0.001 (− 0.008, 0.006)
MVPA	LPA	0.002 (− 0.009, 0.012)	0.006 (− 0.004, 0.016)	0.004 (− 0.007, 0.014)	0.003 (− 0.005, 0.011)	0.003 (− 0.006, 0.012)
MVPA	Sleep	0.001 (− 0.008, 0.009)	0.003 (− 0.005, 0.010)	0.003 (− 0.005, 0.011)	0.000 (− 0.006, 0.007)	0.001 (− 0.001, 0.008)

## Discussion

This study used compositional data analysis to explore the associations between 24-h movement behaviours and domain-specific psychological wellbeing in a sample of Australian secondary school adolescents. We found that greater time spent in LPA, relative to all other behaviours, was positively associated with happiness, whereas greater time spent sedentary relative to the other behaviours was negatively associated with happiness. No significant associations were found between sleep or MVPA relative to the other behaviours and any wellbeing domains.

The positive association between LPA and happiness scores adds to the limited and inconsistent literature on this topic. According to a systematic review, few studies have examined LPA using waist-worn accelerometry in relation to adolescent mental health, with no significant associations observed with depression or negative affect [51]. However, emerging evidence suggests that LPA may support psychological wellbeing in adolescents. For example, LPA interventions such as tai chi or yoga have been associated with reductions in anxiety symptoms [51, 52]. LPA may represent an accessible, attainable and enjoyable forms of activity that facilitates relaxation, casual social interaction, and emotional regulation compared to MVPA [53]. The observed association may reflect the potential of low-intensity movement to promote relaxation [53], thereby supporting wellbeing. In contrast, higher sedentary time was linked to lower happiness, consistent with previous studies highlighting the role of excessive sedentary time for adolescent psychosocial health [54, 55]. A recent compositional meta-analysis in children and adolescents similarly found limited associations between most movement behaviours and social-emotional outcomes, with sleep showing the strongest positive relationship [56]. While we did not observe sleep effects, the positive association between LPA and happiness suggest that compositional associations may vary across specific wellbeing domains.

Notably, MVPA was not positively associated with any EPOCH domains in this sample. While MVPA is often linked to better adolescent mental health [57, 58], our findings suggest that greater MVPA relative to other behaviours may not necessarily mean greater perceived wellbeing. This should be interpreted cautiously given the small sample size and low variability in MVPA, which may have reduced statistical power to detect significant associations [59, 60]. It is also possible that context influenced the findings. For instance, ecological momentary assessment has shown outdoor or social activity tends to be associated with positive affect, whereas indoor, solitary, or obligatory activity may not [61]. Competitive or compulsive forms of MVPA may induce stress or performance pressure, potentially reducing its psychological benefits [18]. This highlights the need to consider not only the quantity but also the context of MVPA when evaluating its relationship with wellbeing outcomes. Moreover, accelerometers cannot capture domain specific MVPA, which may be critical for understanding its relationship with wellbeing. Evidence suggests that recreational or school sport MVPA may have different psychological effects than work or transport related physical activity [62, 63].

The reallocation findings further suggest that reallocating 30 min from sedentary time to LPA was associated with increased happiness, while reallocating time from LPA to sedentary behaviour was associated with reduced happiness. These findings suggest that small, attainable shifts from sedentary to light activity such as encouraging movement during class or breaks [64], may support adolescent psychological wellbeing. However, sedentary behaviour may include screen-based and non-screen activities (e.g., reading or doing homework). These different domains may have different associations with wellbeing as passive screen time (e.g., television watching) may have more detrimental effects on wellbeing than active screen use (e.g., online gaming) [65].

By applying the EPOCH framework, this study provides new insights into how different movement behaviours relate to specific domains of adolescent psychological wellbeing. From a practical perspective, the findings may inform school and community-based interventions aimed at promoting wellbeing. In particular, promoting opportunities for LPA and reducing sedentary time could be considered alongside existing strategies that encourage sleep and MVPA. Further research is needed to better understand associations between not just intensities of movement behaviour across the 24-h period and wellbeing, but also the contexts in which these behaviours occur. Moreover, while wellbeing can fluctuate, particularly in hedonic domains, the EPOCH measure also captures more stable eudaimonic traits. Nevertheless, within-person or momentary designs of both 24-h movement behaviours and wellbeing may offer additional insights [66, 67].

The use of compositional data analysis is a strength, as it accounts for the finite and interdependent nature of time-use data. By using CoDA and time reallocation models, we were able to explore how shifts between behaviours vary the relationships with wellbeing domains. This approach offers insight into how changes in daily routines may be associated with psychological wellbeing in adolescents. A further strength of this study was the use of device-based measures to capture daily movement behaviours, reducing the biases associated with self-reporting [40], and the use of the EPOCH measure enabled the exploration of wellbeing beyond traditional mental illness.

Several limitations should also be noted. As this was an exploratory cross-sectional study with a relatively small sample, causal inference is limited. More than 30% of the consenting sample were excluded due to insufficient accelerometer data, a rate consistent with previous adolescent studies [68, 69], but potentially introducing non-participation bias [70]. However, comparisons between included and excluded participants on wellbeing scores and demographic characteristics revealed no significant differences (Supplementary file, Table 1). The relationship between sleep and wellbeing is often described as non-linear, with both insufficient and excessive sleep linked to poorer outcomes in adolescents. Our analyses modelled sleep effects linearly, as the maximum observed sleep duration in our sample (10.3 h) fell within recommended guidelines, limiting the ability to test potential non-linear associations. Moreover, although reallocations were associated with measurable changes in wellbeing domain scores, there is currently no established threshold for a clinically significant change. Finally, although accelerometer data provides device-based estimates of movement behaviours, it cannot distinguish between contexts or types of activity, or differentiate between sitting and standing postures, which may have influenced our estimates differently [71].

## Conclusions

This study provides evidence linking the daily composition of movement behaviours to domain-specific aspects of adolescent psychological wellbeing. LPA was positively associated with happiness, while higher sedentary time relative to other behaviours was associated with lower happiness. In the reallocation models, shifting 30 min from sedentary time to LPA was associated with higher happiness scores, whereas reallocations involving sleep and MVPA were not statistically significant. These findings suggest that small reductions in sedentary time replaced with light activity may support aspects of adolescent wellbeing. Future research should explore these associations longitudinally and include contextual information about movement behaviours to better understand how changes to daily routines may influence adolescent wellbeing.

## Supplementary Information

Below is the link to the electronic supplementary material.

Supplementary Material 1.

## Data Availability

The datasets used and/or analysed during the current study are available from the corresponding author on reasonable request.

## Abbreviations

WHO: World Health Organisation
EPOCH: Engagement, perseverance, optimism, connectedness, happiness
MVPA: Moderate to vigorous physical activity
LPA: Light physical activity
MPA: Moderate physical activity
VPA: Vigorous physical activity
CoDA: Compositional data analysis
ILR: Isometric log-ratio

## References

[1] Andrews FM, Withey SB. Social Indicators of well-being: America’s perception of life quality. 1 ed. Springer New York, NY; 1976.

[2] Ross DA, Hinton R, Melles-Brewer M, Engel D, Zeck W, Fagan L, et al. Adolescent well-being: a definition and conceptual framework. J Adolesc Health. 2020;67(4):472–6. 32800426 10.1016/j.jadohealth.2020.06.042 PMC7423586

[3] Marquez J, Taylor L, Boyle L, Zhou W, De Neve J. Child and adolescent well-being: global trends, challenges and opportunities. 2024.

[4] Cosma A, Abdrakhmanova S, Taut D, Schrijvers K, Catunda C, Schnohr C. A focus on adolescent mental health and wellbeing in Europe, central Asia and Canada. Health behaviour in school-aged children international report from the. 2021;2022.

[5] Dumuid D, Singh B, Brinsley J, Virgara R, Curtis RG, Brinkman S, et al. Trends in well-being among youth in Australia, 2017–2022. JAMA Netw Open. 2023;6(8):e2330098–e. 37606925 10.1001/jamanetworkopen.2023.30098 PMC10445194

[6] Mesman E, Vreeker A, Hillegers M. Resilience and mental health in children and adolescents: an update of the recent literature and future directions. Curr Opin Psychiatry. 2021;34(6). 10.1097/YCO.0000000000000741 PMC8500371 34433193

[7] Guerra-Bustamante J, León-Del-Barco B, Yuste-Tosina R, López-Ramos VM, Mendo-Lázaro S. Emotional intelligence and psychological Well-Being in adolescents. Int J Environ Res Public Health. 2019;16(10). 10.3390/ijerph16101720 PMC6572191 31100799

[8] Moreira P, Pedras S, Pombo P. Students’ personality contributes more to academic performance than well-being and learning approach-implications for sustainable development and education. Eur J Investig Health Psychol Educ. 2020;10(4):1132–49. 34542440 10.3390/ejihpe10040079 PMC8314314

[9] Kaasbøll J, Sigurdson JF, Skokauskas N, Sund AM. Cohort profile: the youth and mental health study (YAMHS) - a longitudinal study of the period from adolescence to adulthood. PLoS ONE. 2021;16(2):e0247036. 33606731 10.1371/journal.pone.0247036 PMC7895392

[10] Kern ML, Benson L, Steinberg EA, Steinberg L. The EPOCH measure of adolescent well-being. Psychol Assess. 2016;28(5):586–97. 26302102 10.1037/pas0000201

[11] Oberle E, Fan S, Molyneux TM, Ji XR, Brussoni M. Adherence to 24-hour movement guidelines and associations with mental well-being: a population-based study with adolescents in Canada. BMC Public Health. 2025;25(1):749. 40050844 10.1186/s12889-025-21857-7 PMC11884116

[12] Department of Health. Australia’s Physical Activity and Sedentary Behaviour Guidelines and the Australian 24-Hour Movement Guidelines 2019 [Available from: https://www1.health.gov.au/internet/main/publishing.nsf/Content/health-pubhlth-strateg-phys-act-guidelines

[13] Okely AD, Ghersi D, Loughran SP, Cliff DP, Shilton T, Jones RA, et al. A collaborative approach to adopting/adapting guidelines. The Australian 24-hour movement guidelines for children (5–12 years) and young people (13–17 years): an integration of physical activity, sedentary behaviour, and sleep. Int J Behav Nutr Phys Activity. 2022;19(1):2. 10.1186/s12966-021-01236-2 PMC8734238 34991606

[14] Scully M, Gascoyne C, Wakefield M, Morley B. Prevalence and trends in Australian adolescents’ adherence to 24-hour movement guidelines: findings from a repeated National cross-sectional survey. BMC Public Health. 2022;22(1):105. 35033054 10.1186/s12889-021-12387-z PMC8760722

[15] Groves CI, Huong C, Porter CD, Summerville B, Swafford I, Witham B, et al. Associations between 24-h movement behaviors and indicators of mental health and well-being across the lifespan: a systematic review. J Activity Sedentary Sleep Behav. 2024;3(1):9. 10.1186/s44167-024-00048-6 PMC11960375 40217439

[16] Michaela P, Alan PB, Melinda C, Tim C, Rhiannon P, Nigel S, et al. Physical activity and exercise in youth mental health promotion: a scoping review. BMJ Open Sport Exerc Med. 2020;6(1):e000677. 10.1136/bmjsem-2019-000677 PMC7010991 32095272

[17] Kandola A, Ashdown-Franks G, Hendrikse J, Sabiston CM, Stubbs B. Physical activity and depression: towards Understanding the antidepressant mechanisms of physical activity. Neurosci Biobehavioral Reviews. 2019;107:525–39. 10.1016/j.neubiorev.2019.09.040 31586447

[18] Doré I, Sylvester B, Sabiston C, Sylvestre M-P, O’Loughlin J, Brunet J, et al. Mechanisms underpinning the association between physical activity and mental health in adolescence: a 6-year study. Int J Behav Nutr Phys Activity. 2020;17(1):9. 10.1186/s12966-020-0911-5 PMC6993479 32005251

[19] Hoare E, Milton K, Foster C, Allender S. The associations between sedentary behaviour and mental health among adolescents: a systematic review. Int J Behav Nutr Phys Act. 2016;13(1):108. 27717387 10.1186/s12966-016-0432-4 PMC5055671

[20] Rodriguez-Ayllon M, Cadenas-Sánchez C, Estévez-López F, Muñoz NE, Mora-Gonzalez J, Migueles JH, et al. Role of physical activity and sedentary behavior in the mental health of Preschoolers, children and adolescents: A systematic review and Meta-Analysis. Sports Med. 2019;49(9):1383–410. 30993594 10.1007/s40279-019-01099-5

[21] Liu J, Ji X, Pitt S, Wang G, Rovit E, Lipman T, et al. Childhood sleep: physical, cognitive, and behavioral consequences and implications. World J Pediatr. 2024;20(2):122–32. 36418660 10.1007/s12519-022-00647-w PMC9685105

[22] Sampasa-Kanyinga H, Colman I, Goldfield GS, Janssen I, Wang J, Podinic I, et al. Combinations of physical activity, sedentary time, and sleep duration and their associations with depressive symptoms and other mental health problems in children and adolescents: a systematic review. Int J Behav Nutr Phys Act. 2020;17(1):72. 32503638 10.1186/s12966-020-00976-x PMC7273653

[23] Ahmed KR, Horwood S, Khan A. Effects of a School-based physical activity intervention on adolescents’ mental health: a cluster randomized controlled trial. J Phys Activity Health. 2023:1–7. 10.1123/jpah.2023-0062 37611913

[24] Andermo S, Hallgren M, Nguyen T-T-D, Jonsson S, Petersen S, Friberg M, et al. School-related physical activity interventions and mental health among children: a systematic review and meta-analysis. Sports Med - Open. 2020;6(1):25. 32548792 10.1186/s40798-020-00254-x PMC7297899

[25] Mathews A, Gibbons N, Harrison E, Ukoumunne C, Stallard O. A feasibility study to explore the use of digital treatment of sleep as a first-step intervention to improve adolescent mental health. Behav Sleep Med. 2023;21(2):172–84. 35435785 10.1080/15402002.2022.2063866

[26] Bacaro V, Miletic K, Crocetti E. A meta-analysis of longitudinal studies on the interplay between sleep, mental health, and positive well-being in adolescents. Int J Clin Health Psychol. 2024;24(1):100424. 38125984 10.1016/j.ijchp.2023.100424 PMC10730350

[27] Casanova F, O’Loughlin J, Karageorgiou V, Beaumont RN, Bowden J, Wood AR, et al. Effects of physical activity and sedentary time on depression, anxiety and well-being: a bidirectional Mendelian randomisation study. BMC Med. 2023;21(1):501. 38110912 10.1186/s12916-023-03211-z PMC10729457

[28] Buchan MC, Romano I, Butler A, Laxer RE, Patte KA, Leatherdale ST. Bi-directional relationships between physical activity and mental health among a large sample of Canadian youth: a sex-stratified analysis of students in the COMPASS study. Int J Behav Nutr Phys Activity. 2021;18(1):132. 10.1186/s12966-021-01201-z PMC8501578 34627283

[29] Weinert D, Gubin D. The impact of physical activity on the circadian system: benefits for Health, performance and wellbeing. Appl Sci. 2022;12(18):9220.

[30] Giurgiu M, Ebner-Priemer UW. The 24-hour cognitive-affective physical behavior model: a theoretical framework for studying determinants and health consequences of physical activity, sedentary behavior, and sleep. J Activity Sedentary Sleep Behav. 2025;4(1):6.

[31] Edwards MK, Addoh O, Herod SM, Rhodes RE, Loprinzi PD. A conceptual neurocognitive Affect-Related model for the promotion of exercise among obese adults. Curr Obes Rep. 2017;6(1):86–92. 28205157 10.1007/s13679-017-0244-0

[32] Zhu X, Haegele JA, Healy S. Movement and mental health: behavioral correlates of anxiety and depression among children of 6–17 years old in the U.S. Ment Health Phys Act. 2019;16:60–5.

[33] Zhang J, Yang SX, Wang L, Han LH, Wu XY. The influence of sedentary behaviour on mental health among children and adolescents: A systematic review and meta-analysis of longitudinal studies. J Affect Disord. 2022;306:90–114. 35304232 10.1016/j.jad.2022.03.018

[34] Morales-Muñoz I, Gregory AM. Sleep and mental health problems in children and adolescents. Sleep Med Clin. 2023. 10.1016/j.jsmc.2023.01.006 37120167

[35] Chastin SFM, Palarea-Albaladejo J, Dontje ML, Skelton DA. Combined effects of time spent in physical Activity, sedentary behaviors and sleep on obesity and Cardio-Metabolic health markers: A novel compositional data analysis approach. PLoS ONE. 2015;10(10):e0139984. 26461112 10.1371/journal.pone.0139984 PMC4604082

[36] de Faria FR, Barbosa D, Howe CA, Canabrava KLR, Sasaki JE, dos Santos Amorim PR. Time-use movement behaviors are associated with scores of depression/anxiety among adolescents: A compositional data analysis. PLoS ONE. 2022;17(12):e0279401. 36584176 10.1371/journal.pone.0279401 PMC9803290

[37] Duncan MJ, Kuzik N, Silva DAS, Bélanger RE, Carson V, Chaput J-P, et al. Goldilocks days for adolescent mental health: movement behaviour combinations for well-being, anxiety and depression by gender. Ment Health Phys Act. 2024;26:100572.

[38] Fairclough SJ, Tyler R, Dainty JR, Dumuid D, Richardson C, Shepstone L, et al. Cross-sectional associations between 24-hour activity behaviours and mental health indicators in children and adolescents: A compositional data analysis. J Sports Sci. 2021;39(14):1602–14. 33615990 10.1080/02640414.2021.1890351

[39] Curtis RG, Dumuid D, McCabe H, Singh B, Ferguson T, Maher C. The association between 24-hour activity, sedentary and sleep compositions and mental health in Australian adults: a cross-sectional study. J Activity Sedentary Sleep Behav. 2023;2(1):15. 10.1186/s44167-023-00024-6 PMC11960370 40217512

[40] Brenner PS, DeLamater JD. Social desirability bias in Self-reports of physical activity: is an exercise identity the culprit? Soc Indic Res. 2014;117(2):489–504.

[41] Contardo Ayala AM, Lander N, Mazzoli E, Timperio A, Koorts H, Ridgers ND, et al. Protocol of the *TransformUs secondary* schools program: a type II hybrid implementation-effectiveness trial to increase adolescents’ physical activity and reduce sedentary time in secondary schools. BMJ Open. 2025;15(2):e090468. 39929514 10.1136/bmjopen-2024-090468 PMC11815412

[42] Buerger S, Holzer J, Yanagida T, Schober B, Spiel C. Measuring adolescents’ Well-Being in schools: the adaptation and translation of the EPOCH measure of adolescent Well-Being—A validation study. School Mental Health. 2023;15(2):611–26.

[43] Taheri A, Pourshahriari M, Abdollahi A, Hosseinian S. Psychometric assessment of the Persian translation of the EPOCH measure among adolescent girls. Curr Psychol. 2022;41(7):4961–70.

[44] Yusoff SR, Hoesni SM, Rosharudin NA, Muhammad NA. Validity study of the EPOCH measure of adolescent Well-being in Malaysian samples. Psikohumaniora: Jurnal Penelitian Psikologi. 2024;9(1):107–24.

[45] Migueles JH, Rowlands AV, Huber F, Sabia S, van Hees VT. GGIR: a research community–driven open source R package for generating physical activity and sleep outcomes from multi-day Raw accelerometer data. J Meas Phys Behav. 2019;2(3):188–96.

[46] van Hees VT, Gorzelniak L, Dean León EC, Eder M, Pias M, Taherian S, et al. Separating movement and gravity components in an acceleration signal and implications for the assessment of human daily physical activity. PLoS ONE. 2013;8(4):e61691. 23626718 10.1371/journal.pone.0061691 PMC3634007

[47] van Hees VT, Sabia S, Jones SE, Wood AR, Anderson KN, Kivimäki M, et al. Estimating sleep parameters using an accelerometer without sleep diary. Sci Rep. 2018;8(1):12975. 30154500 10.1038/s41598-018-31266-z PMC6113241

[48] Hildebrand M, VT VANH, Hansen BH, Ekelund U. Age group comparability of Raw accelerometer output from wrist- and hip-worn monitors. Med Sci Sports Exerc. 2014;46(9):1816–24. 24887173 10.1249/MSS.0000000000000289

[49] von Rosen P. Analysing time-use composition as dependent variables in physical activity and sedentary behaviour research: different compositional data analysis approaches. J Activity Sedentary Sleep Behav. 2023;2(1):23. 10.1186/s44167-023-00033-5 PMC11960251 40217358

[50] Grgic J, Dumuid D, Bengoechea EG, Shrestha N, Bauman A, Olds T, et al. Health outcomes associated with reallocations of time between sleep, sedentary behaviour, and physical activity: a systematic scoping review of isotemporal substitution studies. Int J Behav Nutr Phys Activity. 2018;15(1):69. 10.1186/s12966-018-0691-3 PMC6043964 30001713

[51] Felez-Nobrega M, Bort-Roig J, Ma R, Romano E, Faires M, Stubbs B, et al. Light-intensity physical activity and mental ill health: a systematic review of observational studies in the general population. Int J Behav Nutr Phys Activity. 2021;18(1):123. 10.1186/s12966-021-01196-7 PMC8444599 34526048

[52] van Woudenberg TJ, Bevelander KE, Burk WJ, Buijzen M. The reciprocal effects of physical activity and happiness in adolescents. Int J Behav Nutr Phys Activity. 2020;17(1):147. 10.1186/s12966-020-01058-8 PMC7678192 33213465

[53] Telford DM, Meiring RM, Gusso S. Moving beyond moderate-to-vigorous physical activity: the role of light physical activity during adolescence. Front Sports Act Living. 2023;Volume 5–2023. 10.3389/fspor.2023.1282482 PMC10652412 38022771

[54] Kuzik N, da Costa BGG, Hwang Y, Verswijveren S, Rollo S, Tremblay MS, et al. School-related sedentary behaviours and indicators of health and well-being among children and youth: a systematic review. Int J Behav Nutr Phys Act. 2022;19(1):40. 35382825 10.1186/s12966-022-01258-4 PMC8979786

[55] Suchert V, Hanewinkel R, Isensee B. Sedentary behavior and indicators of mental health in school-aged children and adolescents: A systematic review. Prev Med. 2015;76:48–57. 25895839 10.1016/j.ypmed.2015.03.026

[56] Bourke M, Wang HFW, Fortnum K, Thomas G, O’Flaherty M, Mulcahy SK, et al. Association between 24-h movement behaviors and mental health in children and adolescents: A systematic review and compositional data Meta-Analysis. Scand J Med Sci Sports. 2025;35(8):e70120. 40827935 10.1111/sms.70120 PMC12363385

[57] Michaela P, Alan PB, Melinda C, Tim C, Rhiannon P, Nigel S, et al. Physical activity and exercise in youth mental health promotion: a scoping review. BMJ Open Sport Exerc Med. 2020;6(1):e000677. 10.1136/bmjsem-2019-000677 PMC7010991 32095272

[58] Spruit A, Assink M, van Vugt E, van der Put C, Stams GJ. The effects of physical activity interventions on psychosocial outcomes in adolescents: A meta-analytic review. Clin Psychol Rev. 2016;45:56–71. 27064552 10.1016/j.cpr.2016.03.006

[59] Schneider M, Dunn A, Cooper D. Affect, Exercise, and physical activity among healthy adolescents. J Sport Exerc Psychol. 2009;31(6):706–23. 20384008 10.1123/jsep.31.6.706 PMC3531994

[60] Schneider M, Schmalbach P. Affective response to exercise and preferred exercise intensity among adolescents. J Phys Activity Health. 2015;12(4):546–52. 10.1123/jpah.2013-0442 PMC4333108 24770461

[61] Bourke M, Hilland TA, Craike M. Contextual influences on the within-person association between physical activity and affect in adolescents: an ecological momentary assessment study. J Behav Med. 2021;44(3):296–309. 33387176 10.1007/s10865-020-00197-4

[62] Bourke M, Hilland TA, Craike M. Domain specific association between physical activity and affect in adolescents’ daily lives: an ecological momentary assessment study. Psychol Health. 2023;38(3):369–88. 34445903 10.1080/08870446.2021.1965603

[63] Teychenne M, Sousa GM, Baker T, Liddelow C, Babic M, Chauntry AJ et al. Domain-specific physical activity and mental health: an updated systematic review and multilevel meta-analysis in a combined sample of 3.3 million people. Br J Sports Med. 2025:bjsports–2025. 10.1136/bjsports-2025-109806 PMC12702480 40903203

[64] Salmon J, Arundell L, Cerin E, Ridgers ND, Hesketh KD, Daly RM, et al. Transform-Us! Cluster RCT: 18-month and 30-month effects on children’s physical activity, sedentary time and cardiometabolic risk markers. Br J Sports Med. 2023;57(5):311–9. 36428089 10.1136/bjsports-2022-105825 PMC9985722

[65] Hallgren M, Nguyen T-T-D, Owen N, Stubbs B, Vancampfort D, Lundin A, et al. Cross-sectional and prospective relationships of passive and mentally active sedentary behaviours and physical activity with depression. Br J Psychiatry. 2020;217:413–9. 30895922 10.1192/bjp.2019.60

[66] Le F, Yap Y, Tung NYC, Bei B, Wiley JF. The associations between daily activities and affect: a compositional isotemporal substitution analysis. Int J Behav Med. 2022;29(4):456–68. 34608593 10.1007/s12529-021-10031-z

[67] Le F, Mattern V, Johansson PJ, Hettiarachchi P, Ebner-Priemer U, Wiley JF, et al. Associations between daily composition of 24 h physical behavior with affective States and working memory. Sci Rep. 2025;15(1):14455. 40281136 10.1038/s41598-025-99266-4 PMC12032417

[68] Burchartz A, Kolb S, Klos L, Schmidt SCE, von Haaren-Mack B, Niessner C, et al. How specific combinations of epoch length, non-wear time and cut-points influence physical activity. German J Exerc Sport Res. 2024;54(2):169–78.

[69] Toftager M, Kristensen PL, Oliver M, Duncan S, Christiansen LB, Boyle E, et al. Accelerometer data reduction in adolescents: effects on sample retention and bias. Int J Behav Nutr Phys Activity. 2013;10(1):140. 10.1186/1479-5868-10-140 PMC3880051 24359480

[70] Antoun C, Wenz A. Nonparticipation bias in Accelerometer-Based studies and the use of propensity scores. Social Sci Comput Rev. 2024:08944393241254463. 10.1177/08944393241254463 PMC12560259 41164738

[71] Li YM, Hachenberger J, Lemola S. The role of the context of physical activity for its association with affective Well-Being: an experience sampling study in young adults. Int J Environ Res Public Health. 2022;19(17). 10.3390/ijerph191710468 PMC9518586 36078182

