# Supplementary material for: Associations between 24-hour movement behaviours and psychological wellbeing in adolescents using compositional data analysis
Source: J Act Sedentary Sleep Behav. 2025 Dec 20;4:21. doi: 10.1186/s44167-025-00094-8 (PMC12750839; doi:10.1186/s44167-025-00094-8)
Supplement: Supplementary file 1 — Supplementary Material 1. [file 44167_2025_94_MOESM1_ESM.docx]

**Table 1. Descriptive characteristics of the sample**

|  | **N=124** | **N=69** | **p-values** |
| --- | --- | --- | --- |
| **Age (Mean (SD))** | 14.8 (1.0) | 14.7 (1.0) | 0.234 |
| **Gender** |  |  | 0.559 |
| Male | 61 | 37 |  |
| Female | 57 | 25 |  |
| Other | 2 | 2 |  |
| Prefer not to say | 4 | 5 |  |
| **Year** |  |  | 0.952 |
| Year 7 | 64 | 37 |  |
| Year 8 | 37 | 22 |  |
| Year 9 | 22 | 10 |  |
| Year 10 | 1 | 0 |  |
| **Country of birth** |  |  | 0.627 |
| Australia | 99 | 60 |  |
| Other | 25 | 9 |  |
| **Language** |  |  | 0.854 |
| English | 101 | 61 |  |
| Other | 23 | 8 |  |
| **EPOCH (mean)** | Range (0-5) |  |  |
| Engagement | 3.39 | 3.32 | 0.840 |
| Perseverance | 3.62 | 3.53 | 0.844 |
| Optimism | 3.61 | 3.55 | 0.678 |
| Connectedness | 4.35 | 4.27 | 0.740 |
| Happiness | 3.92 | 3.79 | 0.423 |

Descriptive statistics of the proportion of time spent in the four behaviours are detailed in Table 2.

**Table 2.** **Descriptive statistics of time in movement behaviours**

| **Movement behaviour** | **Compositional mean (n=124)** | **Compositional mean (n=69)** | **Minutes/day – mean (n=124)** | **Minutes/day – mean (n=69)** |
| --- | --- | --- | --- | --- |
| Sleep | 0.33 | 0.33 | 473.0 | 474.4 |
| SB | 0.47 | 0.47 | 680.9 | 677.8 |
| LPA | 0.18 | 0.17 | 250.7 | 252.1 |
| MVPA | 0.02 | 0.02 | 35.3 | 35.7 |

LPA light-intensity physical activity, MVPA moderate- to vigorous-intensity physical activity, SB sedentary time. Weighted averages have been use

**Table 3. Component linear regression of 24-h activity behaviour time distribution and EPOCH (n=69)**

| **Activity Behaviour** | **Engagement** |  | **Perseverance** |  | **Optimism** |  | **Connectedness** |  | **Happiness** |  |
| --- | --- | --- | --- | --- | --- | --- | --- | --- | --- | --- |
|  | β | p | β | p | β | p | β | p | β | p |
| **ILR 1** |  |  |  |  |  |  |  |  |  |  |
| (Sleep+SB+LPA) vs MVPA | -0.150 | 0.712 | -0.508 | 0.194 | -0.354 | 0.362 | 0.121 | 0.714 | -0.302 | 0.422 |
| (Sleep+SB) vs LPA | -0.072 | 0.863 | 0.135 | 0.736 | -0.176 | 0.660 | 0.308 | 0.365 | 0.544 | 0.161 |
| Sleep vs SB | 0.069 | 0.653 | 0.097 | 0.511 | -0.044 | 0.763 | -0.150 | 0.233 | 0.038 | 0.780 |
| **ILR 2** |  |  |  |  |  |  |  |  |  |  |
| (SB+LPA+MVPA) vs Sleep | 0.012 | 0.976 | 0.371 | 0.340 | 0.024 | 0.949 | 0.206 | 0.530 | 0.622 | 0.097 |
| (SB+LPA) vs MVPA | 0.121 | 0.567 | 0.216 | 0.288 | 0.089 | 0.657 | -0.224 | 0.186 | 0.034 | 0.861 |
| SB vs LPA | 0.133 | 0.735 | 0.319 | 0.401 | 0.387 | 0.305 | -0.194 | 0.545 | -0.023 | 0.949 |
| **ILR 3** |  |  |  |  |  |  |  |  |  |  |
| (Sleep+LPA+MVPA) vs SB | 0.098 | 0.759 | 0.001 | 0.996 | 0.065 | 0.831 | -0.300 | 0.251 | -0.281 | 0.344 |
| (LPA+MVPA) vs Sleep | 0.102 | 0.779 | 0.157 | 0.653 | 0.343 | 0.325 | -0.205 | 0.489 | -0.207 | 0.539 |
| MVPA vs LPA | -0.111 | 0.755 | -0.511 | 0.139 | -0.191 | 0.576 | -0.003 | 0.990 | -0.516 | 0.120 |
| **ILR 4** |  |  |  |  |  |  |  |  |  |  |
| (Sleep+SB+MVPA) vs LPA | 0.039 | 0.881 | 0.135 | 0.591 | 0.264 | 0.293 | -0.027 | 0.899 | -0.038 | 0.874 |
| (Sleep+MVPA) vs SB | -0.150 | 0.660 | -0.509 | 0.124 | -0.257 | 0.434 | 0.124 | 0.656 | -0.371 | 0.243 |
| Sleep vs MVPA | -0.091 | 0.828 | 0.095 | 0.814 | 0.151 | 0.707 | 0.341 | 0.321 | 0.499 | 0.201 |

Abbreviations: SB, sedentary time; LPA, light physical activity; MVPA, moderate-to-vigorous physical activity. Adjusted for age and gender and clustering of schools.
